# Assessment of the gradient diffusion method for fosfomycin susceptibility testing in *Staphylococcus* spp. and *Enterococcus* spp. isolated from the urine of companion dogs in Thailand

**DOI:** 10.14202/vetworld.2023.2497-2503

**Published:** 2023-12-25

**Authors:** Nattha Jariyapamornkoon, Pongthai Boonkam, Nipattra Suanpairintr

**Affiliations:** 1 Department of Agricultural Technology, Faculty of Science and Technology, Thammasat University, Pathum Thani, Thailand; 2 Veterinary Diagnostic Laboratory, Faculty of Veterinary Science, Chulalongkorn University, Bangkok, Thailand; 3 Department of Pharmacology, Faculty of Veterinary Science, Chulalongkorn University, Bangkok, Thailand; 4 Center of Excellence for Food and Water Risk Analysis (FAWRA), Department of Veterinary Public Health, Faculty of Veterinary Science, Chulalongkorn University, Bangkok, Thailand

**Keywords:** dogs, *Enterococcus* spp, fosfomycin, gradient diffusion method, *Staphylococcus* spp

## Abstract

**Background and Aim:**

The agar dilution method is the approved method for determining the minimum inhibitory concentration (MIC) in fosfomycin susceptibility testing, whereas the broth dilution method is not recommended. This study aimed to investigate the potential of the gradient diffusion method as a more convenient alternative to agar dilution method for MIC evaluation, particularly for the susceptibility testing of *Staphylococcus* spp. and *Enterococcus* spp. to fosfomycin.

**Materials and Methods:**

A total of 194 isolates of *Staphylococcus* spp. and *Enterococcus* spp. were collected from urine samples of dogs diagnosed with bacterial cystitis. Bacterial identification and susceptibility to multiple antibiotics were tested using the Vitek 2 automated system. The susceptibility to fosfomycin was compared between agar dilution (reference method) and the gradient diffusion method. We assessed the agreement rates and errors between the two approaches by analyzing the MIC data.

**Results:**

*Staphylococcus pseudintermedius* (98.7%) and *Enterococcus faecalis* (80.0%) exhibited high fosfomycin susceptibility rates, whereas *Enterococcus faecium* exhibited a lower susceptibility rate (38.5%). The gradient diffusion method demonstrated unacceptably low essential agreement (EA) rates (>90%) but acceptable categorical agreement (CA) rates (≥ 90%) for *S. pseudintermedius* (83.54% EA and 97.47% CA) and coagulase-negative staphylococci (CoNS) such as *Staphylococcus chromogenes*, *Staphylococcus hominis*, and *Staphylococcus simulans* (85.00% EA and 95.00% CA). *Enterococcus* spp. had an acceptable EA of 93.75%, but an unacceptably low CA rate of 82.81%, with a minor error rate of 17.19%. No significant errors were observed for *Staphylococcus* and *Enterococcus* spp.

**Conclusion:**

The gradient diffusion method reliably determines MICs and interpretative breakpoints (S, I, R) for *S. pseudintermedius*. However, its applicability to CoNS and enterococci may be limited due to unacceptable errors.

## Introduction

Fosfomycin is a broad-spectrum bactericidal drug. This drug does not undergo any metabolic process and is excreted in its active form through the kidneys [1, 2]. The high concentrations of fosfomycin in urine make it a favorable choice for treating uncomplicated urinary tract infections (UTIs) in humans [3, 4]. However, fosfomycin has not yet been approved for veterinary use in many countries, including the European Union, and its administration to individual companion animals is restricted to exceptional circumstances due to concerns related to public health and the growing problem of antimicrobial resistance [5, 6]. Therefore, there are limited data on the use of fosfomycin in animals. Fosfomycin has been suggested as an alternative antibacterial drug for treating bacterial cystitis in dogs when other antibacterial drugs are ineffective or unavailable [7, 8].

Bacterial cystitis is widely recognized as one of the most common diseases among dogs. In Thailand, *Staphylococcus* spp. and *Escherichia coli* are the main uropathogens of these infections [9, 10]. The antimicrobial resistance of methicillin-resistant *Staphylococcus pseudintermedius* (MRSP) is of particular concern. Several studies have indicated that MRSP strains display multidrug resistance (MDR) to three or more antibacterial classes [11, 12]. Enterococci commonly cause UTIs and are naturally resistant to many antibacterial drugs [13, 14]. Therefore, the susceptibility data of *Staphylococcus* spp. and *Enterococcus* spp., which are the main Gram-positive uropathogens in dogs, may provide supportive information for the clinical treatment of fosfomycin.

Based on the Clinical Laboratory Standards Institute (CLSI) and the European Committee on Antimicrobial Susceptibility Testing (EUCAST), the agar dilution method is the approved method for minimum inhibitory concentration (MIC) measurement in the susceptibility testing of fosfomycin, whereas the broth dilution method is not recommended [15, 16]. Automated susceptibility testing should therefore be avoided [17]. However, the agar dilution method has certain disadvantages, such as being time-consuming, laborious, and inconvenient for routine laboratory work. Alternatively, gradient diffusion method may offer a more convenient practice for determining MIC values, but its reliability needs to be evaluated. The agreement between agar dilution and gradient diffusion methods has been extensively studied in bacteria such as *Klebsiella pneumoniae* and *E. coli* [17–19]. However, it does not agree with Gram-positive bacteria, especially in animals, and remains poorly explored. To address this research gap, the present study focuses on investigating *Staphylococcus* spp. and *Enterococcus* spp. found in dog urine.

The main objectives of this study were to determine the susceptibility of these bacteria to fosfomycin and other antibacterial drugs and to evaluate the reliability of the gradient diffusion method compared to the reference method for susceptibility testing.

## Materials and Methods

### Ethical approval

This study does not require ethical approval. All urine samples were obtained from the Veterinary Diagnostic Laboratory of the Faculty of Veterinary Science, Chulalongkorn University, Bangkok, Thailand.

### Study period and location

This study was conducted from July to December 2022. The samples were processed at the Faculty of Veterinary Science, Chulalongkorn University, Bangkok, Thailand.

### Bacterial identification

A total of 194 *Staphylococcus* spp. and *Enterococcus* spp. isolates were obtained from the Veterinary Diagnostic Laboratory of the Faculty of Veterinary Science, Chulalongkorn University, Bangkok, Thailand. Bacterial samples were isolated from urine samples of dogs diagnosed with bacterial cystitis at the Small Animal Teaching Hospital of Veterinary Medicine of Chulalongkorn University from 2018 to 2021. Bacterial isolates were identified using a Vitek 2 automated system equipped with Gram-positive identification cards (ID-GP card) (BioMerieux, Marcy L’Étoile, France). For further analysis, all bacterial samples were stored in 30% glycerol storage media at −80°C.

### Susceptibility testing to fosfomycin using agar dilution method

Agar dilution was performed in accordance with the CLSI standard for determining the MIC of fosfomycin [16, 20]. The inoculum was cultured on Mueller–Hinton agar (Difco, Franklin Lakes, NJ, USA) supplemented with 25 mg/L glucose-6-phosphate (Sigma-Aldrich, Saint Louis, MO, USA) plates containing 0.125–256 mg/L fosfomycin (Sigma-Aldrich) using a 48-pin replicator (Sigma-Aldrich). The final inoculum concentration was approximately 1 × 10^4^ colony forming unit/spot. All isolate analyses were performed in triplicate. Quality control testing was conducted by examining the reference strains of *Staphylococcus aureus* ATCC 29213 and *Enterococcus faecalis* ATCC 29212 [16]. The lowest concentration of fosfomycin-inhibited visible colonies was recorded and interpreted based on MIC breakpoints [15, 16].

### Susceptibility testing to fosfomycin using gradient diffusion method

The gradient diffusion technique (Liofilchem, Roseto degli Abruzzi, Italy) was performed according to the manufacturer’s instructions [21]. Isolated bacterial colonies were suspended in a 0.85% sodium chloride solution to achieve a McFarland standard turbidity of 0.5. The prepared inoculum was spread onto Mueller–Hinton II agar (MHA) using a sterile cotton swab. A gradient diffusion strip ranging from 0.016 to 256 mg/L supplemented with glucose-6-phosphate (G6P) was placed onto the MHA surface before incubation at 37°C for 16–20 h or extended for up to 48 h for slow-growing bacteria. We recorded and interpreted the MIC strip’s scale that intersected the inhibition zone according to MIC breakpoints [15, 16]. All of the isolates were tested in triplicate. *Staphylococcus aureus* ATCC 29213 and *E. faecalis* ATCC 29212 [21] were used as quality control isolates.

### Interpretive criteria for MIC of fosfomycin

The EUCAST criteria were applied with MIC breakpoints for *Staphylococcus* spp. as follows: susceptible (S) 32 mg/L and resistance (R) >32 mg/L [15]. The CLSI criteria for *E. faecalis* were implemented with MIC breakpoints: S <64 mg/L, intermediate (I) 128 mg/L, and R >256 mg/L [16]. In the absence of approved fosfomycin breakpoints for *E. faecium*, the MIC breakpoints for *E. faecalis* were utilized to interpret the susceptibility of *E. faecalis*, following a similar approach as in a previous study [19].

### Antibacterial susceptibility testing of other antibacterial drugs

Bacterial isolates were tested using a Vitek 2 automated system with Gram-positive Veterinary Susceptibility Test Cards (GP 76) (BioMerieux). The cards included assessments for various antibacterial drugs, including amoxicillin/clavulanic acid (2–32 mg/L), oxacillin (0.5–4 mg/L), cephalothin (2–32 mg/L), cefovecin (0.5–8 mg/L), cefpodoxime (1–8 mg/L), gentamicin (0.5–16 mg/L), enrofloxacin (0.5–4 mg/L), marbofloxacin (0.5–4 mg/L), tetracycline (1–16 mg/L), nitrofurantoin (16–512 mg/L), vancomycin (0.5–32 mg/L) and trimethoprim/sulfamethoxazole (20 (1/19) – 320 (16/304) mg/L). The susceptibility of each antibacterial drug was interpreted based on its MIC breakpoints [22].

### Comparative analysis of susceptibility testing methods

We analyzed the degree of agreement and error between the MICs obtained using agar dilution (reference) and gradient diffusion methods. The analysis focused on the following parameters: (1) the essential agreement (EA) refers to the MIC of gradient diffusion method equal to or within ±1 dilution of the reference method MIC, (2) the categorical agreement (CA) refers to the agreement of susceptibility categories (S, I, R) between the gradient dilution method and the reference method, (3) major errors (ME) refer to the result of gradient diffusion was resistant but the result of reference method was susceptible, (4) very major errors (VMEs) refer to the result of gradient diffusion was susceptible but the result of reference method was resistant, (5) minor error (mE) refers to when the result of gradient diffusion was susceptible or resistant but the result of reference method was intermediate, or when the result of gradient diffusion was intermediate but the result of reference method was susceptible or resistant [19, 23].

On the basis of CLSI standards, an acceptable rate of >90% for the EA and the CA should be adopted. The acceptable ME and VME should be <3.0% [23].

## Results

### Bacterial identification

*Staphylococcus pseudintermedius* was the predominant bacterial isolate in this study, accounting for 40.7% (n=79/194) of the total isolate. Methicillin-sensitive *Staphylococcus pseudintermedius* (MSSP) and MRSP were 16.0% (n=31/194) and 24.7% (n=48/194), respectively, in this category. Among coagulase-negative staphylococci (CoNS), *Staphylococcus chromogenes* (8.8%, n=17/194), *Staphylococcus hominis*, and *Staphylococcus warneri* each accounted for 3.6% (n=7/194). *E. faecium* (20.1%, n=39/194) and *E. faecalis* (12.9%, n=25/194) were identified as *Enterococcus* isolates.

### Susceptibility to fosfomycin

The agar dilution technique showed that 95.38% of *Staphylococcus* spp. strains were susceptible to fosfomycin, with *S. pseudintermedius*, including MRSP, exhibiting high susceptibility rates above 97% (Table-1). CoNS, including *S. chromogenes*, *S. hominis*, *Staphylococcus simulans*, *Staphylococcus auricularis, Staphylococcus haemolyticus*, and *E. faecalis*, demonstrated susceptibility rates above 80%. Regarding the gradient diffusion method, the susceptibility rates observed for MSSP, *S. aureus*, and *E. faecalis* (Table-2) were similar to those obtained from the agar dilution technique. However, the susceptibility rates for MRSP, CoNS, and *E. faecium* were lower (95.4%, 87.5%, and 33.3%, respectively) than the agar dilution method (97.9%, 90.0%, and 38.5%, respectively).

**Table-1:** Distribution of MICs and the susceptibility rates to fosfomycin (FOS) using agar dilution method.

Bacterial isolates	n (194)	MIC of FOS (mg/L)												Susceptibility to FOS		

		0.125	0.25	0.5	1	2	4	8	16	32	64	128	256	>256	n	S%
CoPS	90														88	97.8
*S. pseudintermedius*	79														78	98.7
MSSP	31	8	14	4	2	2	1								31	100.0
MRSP	48	15	17	10	2	1	1			1	1				47	97.9
*S. aureus*	11		2	6	1	1								1	10	90.9
CoNS	40														36	90.0
*S. chromogenes*	17		2	5	2				6		2				15	88.2
*S. hominis*	7		2	1	1				1	2					7	100.0
*S. warneri*	7			1		1				3	2				5	71.4
*S. simulans*	5			2	1				1	1					5	100.0
*S. auricularis*	2							1	1						2	100.0
*S. haemolyticus*										2					2	100.0
*Enterococcus* spp.	64														36	56.3
*E. faecalis*	25									10	10	5			20	80.0
*E. faecium*	39									7	8	21	3		15	38.5

MIC=Minimum inhibitory concentration, CoPS=Coagulase-positive staphylococci, CoNS=Coagulase-negative staphylococci, MSSP=Methicillin-sensitive *Staphylococcus pseudintermedius,* MRSP=Methicillin-resistance *Staphylococcus pseudintermedius.* No color area=Susceptible (S), Light gray area=Intermediate (I), Dark gray color area=Resistance (R). *S. chromogenes=Staphylococcus chromogenes*, *S. hominis=Staphylococcus hominis*, *S. warneri=Staphylococcus warneri*, *S. simulans=Staphylococcus simulans*, *S. auricularis=Staphylococcus auricularis*, *S. haemolyticus=Staphylococcus haemolyticus*, *S. pseudintermedius=Staphylococcus pseudintermedius, S. aureus=Staphylococcus aureus*, *E. faecalis=Enterococcus faecalis*, *E. faecium=Enterococcus faecium*

**Table-2:** Distribution of MICs and the susceptibility rates to fosfomycin (FOS) using gradient diffusion method.

Bacterial isolates	n (194)	MIC of FOS (mg/L)												Susceptibility to FOS		

		0.125	0.25	0.5	1	2	4	8	16	32	64	128	256	>256	n	S%
CoPS	90														87	96.7
*S. pseudintermedius*	79														77	97.5
MSSP	31	2	6	12	3	5	1	2							31	100
MRSP	48	4	15	14	7	4	2				2				46	95.4
*S. aureus*	11		1	3	2	3	1							1	10	90.9
CoNS	40														35	87.5
*S. chromogenes*	17		1	2	4	2			2	3	1			2	14	82.4
*S. hominis*	7			3		1				3					7	100.0
*S. warneri*	7				1		1			2	1	2			4	57.1
*S. simulans*	5				1	1	1			2					5	100.0
*S. auricularis*	2								1	1					2	100.0
*S. haemolyticus*	2									2					2	100.0
*Enterococcus* spp.	64														33	51.6
*E. faecalis*	25									7	13	3	2		20	80.0
*E. faecium*	39									2	11	14	7	5	13	33.3

MIC=Minimum inhibitory concentration, CoPS=Coagulase-positive staphylococci, CoNS=Coagulase-negative staphylococci, MSSP=Methicillin-sensitive *Staphylococcus pseudintermedius,* MRSP=Methicillin-resistance *Staphylococcus pseudintermedius*. No color area=Susceptible (S), Light gray area=Intermediate (I), Dark gray color area=Resistance (R). *S. chromogenes=Staphylococcus chromogenes*, *S. hominis=Staphylococcus hominis*, *S. warneri=Staphylococcus warneri*, *S. simulans=Staphylococcus simulans*, *S. auricularis=Staphylococcus auricularis*, *S. haemolyticus=Staphylococcus haemolyticus*, *S. pseudintermedius=Staphylococcus pseudintermedius, S. aureus=Staphylococcus aureus*, *E. faecalis=Enterococcus faecalis*, *E. faecium=Enterococcus faecium*

### Antibacterial susceptibility to other antibacterial drugs

Among MRSP isolates, the susceptibility rate to fosfomycin (97.9%) was lower than that of vancomycin and nitrofurantoin (100%), but higher than that of amoxicillin/clavulanic acid (64.6%), trimethoprim/sulfamethoxazole (39.6%), and fluoroquinolones (10.4%) (Table-3). The susceptibility to amoxicillin/clavulanic acid (82.5%) was lower than that to fosfomycin (90.0%) in CoNS isolates. On the other hand, *E. faecalis* isolates exhibited a higher susceptibility to amoxicillin/clavulanic acid (96%) than to fosfomycin (80.0%). The susceptibility rates of *E. faecium* isolates to fosfomycin and nitrofurantoin were similar (38.5%), whereas the susceptibility rates to amoxicillin and clavulanic acid were very low (15.4%). All isolates of *Enterococcus* spp. were susceptible to vancomycin; no resistance was observed.

**Table-3:** Susceptibility to other antibacterial drugs.

Bacterial isolates	Susceptibility (%S)											

	AMC	OXA*	CEF	CPD	CFV	GEN	ENR	MRB	VAN	TET	NIT	SXT
*Staphylococcus* spp.												
*S. pseudintermedius*												
MSSP	100.0	100.0	100.0	100.0	100.0	58.1	58.1	58.1	100.0	29.0	100.0	48.4
MRSP	64.6	0.0	75.0	31.3	35.4	29.2	10.4	10.4	100.0	0.0	100.0	39.6
*S. aureus*	100.0	90.9	90.9	90.9	90.9	90.9	9.1	9.1	100.0	0.0	100.0	81.8
CoNS	82.5	90.0	92.5	85.0	87.5	95.0	72.5	72.5	100.0	70.0	100.0	75.0
*Enterococcus* spp.												
*E. faecalis*	96.0	NA	NA	NA	NA	NA	NA	NA	100.0	24.0	100.0	NA
*E. faecium*	15.4	NA	NA	NA	NA	NA	NA	NA	100.0	7.7	38.5	NA

* OXA was used as a surrogate for methicillin in susceptibility testing, CoNS=Coagulase-negative staphylococci, NA=Not available, AMC=amoxicillin/clavulanic acid, OXA=Oxacillin, CEF=Cephalothin, CPD=Cefpodoxime, CFV=Cefovecin, GEN=Gentamicin, ENR=Enrofloxacin, MRB=Marbofloxacin, VAN=Vancomycin, TET=Tetracycline, NIT=Nitrofurantoin and SXT=Trimethoprim/sulfamethoxazole. *S. pseudintermedius=Staphylococcus pseudintermedius, S. aureus=Staphylococcus aureus*, *E. faecalis=Enterococcus faecalis*, *E. faecium=Enterococcus faecium*

### Comparative analysis of susceptibility testing methods

The MIC_50_ and MIC_90_ values of fosfomycin obtained from agar dilution and gradient diffusion methods were similar or within ±1 doubling dilution for *S. pseudintermedius*, CoNS, and *Enterococcus* spp. (Table-4). The EA between the gradient dilution and agar dilution methods for *Enterococcus* spp. achieved an acceptable rate (90%), whereas it was below 90% for *S. pseudintermedius* and CoNS. Both *S. pseudintermedius* and CoNS achieved an acceptable rate of CA (90%), whereas *Enterococcus* spp. had an unacceptably low CA. *Staphylococcus pseudintermedius* and *Enterococcus* spp. exhibited a ME rate within an acceptable range (3%), whereas CoNS showed unacceptably high ME rates. No significant errors were identified in *Staphylococcus* spp. and *Enterococcus* spp. Minor errors were observed exclusively in *Enterococcus* spp. testing.

**Table-4:** Comparative analysis of susceptibility testing methods.

Bacterial isolates Testing methods	n	MIC (mg/L)			MIC agreement and error (%)				
	
		MIC range	MIC_50_	MIC_90_	EA	CA	ME	VME	mE
*S. pseudintermedius*	79								
Agar dilution		0.125–64	0.25	1					
Gradient diffusion		0.125–64	0.5	2	83.54 (66/79)	97.47 (77/79)	1.28 (1/78)	0.00	0.00
CoNS	40								
Agar dilution		0.25–64	16	32					
Gradient diffusion		0.25–>256	16	64	85.00 (34/40)	95.00 (38/40)	5.56 (2/36)	0.00	0.00
*Enterococcus* spp.	64								
Agar dilution		32–256	64	128					
Gradient diffusion		32–>256	64	256	93.75 (60/64)	82.81 (53/64)	0.00	0.00	17.19 (11/64)

MIC=Minimum inhibitory concentration, CoNS=Coagulase-negative staphylococci, EA=Essential agreement, CA=Categorical agreement, ME=Major errors, VME=Very major errors, mE=Minor error, *S. pseudintermedius=Staphylococcus pseudintermedius*, CoNS=Coagulase-negative staphylococci

## Discussion

Staphylococcus *pseudintermedius* has been frequently identified as the predominant uropathogen causing UTIs in dogs across many countries, including Thailand [9, 24, 25]. MRSP isolates display MDR to three or more different antibacterial classes [11, 12]. Therefore, antimicrobial resistance in *S. pseudintermedius* poses a significant challenge in terms of treatment options for dogs. Coagulase-negative staphylococci are typically considered opportunistic or contaminant pathogens, but they have also been found to be causative agents of infections [26, 27].

In the present study, *Staphylococcus* spp. isolates, including MRSP, displayed high susceptibility rates to fosfomycin. These findings are consistent with a previous study that reported an approximately 84% susceptibility rate in MRSP from dogs, with an MIC_50_ of 0.125 mg/L [28]. Although CoNS isolates showed a 90% susceptibility rate to fosfomycin, approximately 20% of CoNS isolates (n=8/40) exhibited a MIC of 32 mg/L, which closely approached the interpretative breakpoints of S 32 and R > 32 mg/L [16]. Therefore, the use of fosfomycin for CoNS may require intensive monitoring for developing resistance. With regard to *Enterococcus* spp., we observed a high susceptibility rate of 80% for *E. faecalis*, which is consistent with the results reported for humans (94.4%) [29]. However, regarding *E. faecium*, only 38.5% of samples were susceptible to fosfomycin. A previous study indicated that more than 90% of *Enterococcus* spp. isolated from dog wounds and dermatitis are resistant to fosfomycin [30]. These results suggest that fosfomycin may be a suitable treatment for *E. faecalis* but not for *E. faecium*, emphasizing the importance of bacterial identification and susceptibility testing before fosfomycin administration.

Our results indicate that *S. pseudintermedius* isolates exhibit low susceptibility to trimethoprim, sulfamethoxazole, and fluoroquinolones. In addition, MRSP isolates displayed low susceptibility to amoxicillin/clavulanic acid (64.6%), which is a recommended empirical drug for bacterial cystitis in dogs, as well as trimethoprim/sulfamethoxazole [31]. These findings are consistent with the previous studies reporting low susceptibility rates of MRSP strains to amoxicillin/clavulanic acid (35%) [32] and high resistance rates to enrofloxacin (38%) [33]. According to CLSI guidelines, MRSP isolates are generally resistant to all beta-lactam antibacterial drugs except ceftaroline [16]. Considering the high susceptibility rates to fosfomycin (>97%) observed in this study, fosfomycin may be a reasonable alternative antibiotic for *S. pseudintermedius*, including MRSP strains.

The susceptibility rate of *E. faecalis* to amoxicillin/clavulanic acid (96%) was consistent with that reported in the previous studies (95%) [34]. *Enterococcus faecium* isolates from canine UTIs have been reported to exhibit high resistance rates to ampicillin (67.9%), enrofloxacin (91.8%), and nitrofurantoin (90.9%) [13]. *Enterococcus* spp. is known to be intrinsically resistant to various antibacterial drugs [14], with *E. faecium* isolates being more resistant than *E. faecalis* [35]. Therefore, selecting appropriate antibacterial drugs for E. faecium infection may be difficult.

While the use of gradient diffusion strips offers a convenient alternative for measuring MIC, several studies have reported low agreement between MIC results obtained through agar dilution (reference method) and other methods, particularly in *K. pneumoniae* and *E. coli* [17, 18]. The presence of colonies on the agar makes reading difficult and may affect the accuracy of the results [17]. On the other hand, the use of E test to determine MICs in *S. aureus* and *E. faecalis* isolates from humans has shown good concordance with the agar dilution method, as reported in early publications [19, 36]. At present, however, there is no available data on *S. pseudintermedius*.

In the present study, the fosfomycin susceptibility rate of *S. pseudintermedius* obtained using the gradient diffusion method decreased by approximately 1% compared to that obtained using the agar dilution method. The EA rate between these two methods is unacceptably low (83.54%). However, the CA and ME rates fell within acceptable ranges (≥90% and ≤3%, respectively). These findings are consistent with those of an earlier study on *S. aureus*, where the EA, CA, and ME rates were 84.1%, 98.7%, and 1.3%, respectively [19]. The low EA rate suggests that reporting MIC values alone without interpreting them as S, I, or R using the gradient diffusion method may not be appropriate [19]. Regarding CoNS, the agreement results were similar to those of *S. pseudintermedius* (unacceptable EA and acceptable CA), but the ME rate was unacceptably high (5.56%, n=2). The two isolates that exhibited ME results were identified as *S. chromogenes* and *S. warneri*. These findings emphasize the need to exercise caution when interpreting the MIC values obtained using the gradient diffusion method for CoNS.

With regard to *Enterococcus* spp., the study showed an acceptable EA rate; however, the CA rates exceeded the acceptable range (>90%) with a relatively high rate of MEs (17.19%). These results are consistent with those of a previous study on *E. faecalis*, which reported rates of 98.5% for EA, 81.2% for CA, and 18.8% for mE [19]. Unsatisfactory CA and mE rates indicate the importance of interpreting MIC values obtained by gradient diffusion for *Enterococcus* spp. cautiously.

This study has certain limitations. The small number of bacterial samples used in this study may have affected the accuracy of the percentage data. Therefore, agreements and errors were evaluated between different methods using groups of bacteria rather than individual species. In the assessment study, *S. aureus* isolates were not included alongside *S. pseudintermedius* as coagulase-positive staphylococci because *S. pseudintermedius*, the main uropathogen in dogs, was the primary focus of this study. In addition, the interpretation of susceptibility to fosfomycin was based on human guidelines since there are currently no MIC breakpoints for veterinary use.

## Conclusion

The high susceptibility rates of *Staphylococcus* spp. and *E. faecalis* to fosfomycin indicate the efficacy of fosfomycin as an alternative antibiotic for the treatment of canine bacterial cystitis caused by susceptible strains. However, the low susceptibility rate of *E. faecium* suggests that fosfomycin may not be effective against this pathogen. Minimum inhibitory concentration determination in *S. pseudintermedius* with interpretative breakpoints (S, I, and R) proved reliable using the gradient diffusion method. In addition, caution should be exercised when using this method for CoNS and *Enterococcus* spp. due to the presence of unacceptable errors.

## Authors’ Contributions

NJ: Conceptualization, formal analysis, methodology, software, validation, investigation, visualization, project administration, writing–original draft, writing–review, and editing. PB: Methodology. NS: Conceptualization, formal analysis, resources, supervision, and project administration. All authors have read, reviewed, and approved the revised manuscript.
